# Hypobaric hypoxia affects gut microbiota of rats through affected community assembly, reduced network resilience, and metabolic reprogramming

**DOI:** 10.1093/femsec/fiag039

**Published:** 2026-04-16

**Authors:** Xinyang Chen, Yihong Wang, Jianzhuo Feng, Huiqing Chen, Baohui Yao, Fuxin Li, Quanyu Yang, Jiapeng Qu

**Affiliations:** Qinghai Provincial Key Laboratory of Animal Ecological Genomics, Northwest Institute of Plateau Biology, Chinese Academy of Sciences, Qinghai 810008, China; University of Chinese Academy of Sciences, Beijing 100049, China; Qinghai Provincial Key Laboratory of Animal Ecological Genomics, Northwest Institute of Plateau Biology, Chinese Academy of Sciences, Qinghai 810008, China; University of Chinese Academy of Sciences, Beijing 100049, China; School of Life Science, Qinghai Normal University, Xining, Qinghai 810008, China; Qinghai Provincial Key Laboratory of Animal Ecological Genomics, Northwest Institute of Plateau Biology, Chinese Academy of Sciences, Qinghai 810008, China; University of Chinese Academy of Sciences, Beijing 100049, China; Qinghai Provincial Key Laboratory of Animal Ecological Genomics, Northwest Institute of Plateau Biology, Chinese Academy of Sciences, Qinghai 810008, China; University of Chinese Academy of Sciences, Beijing 100049, China; Department of Basic Medicine, Qinghai University Medical College, Xining, Qinghai 810016, China; Research Center for High Altitude Medicine, Qinghai University Medical College, Xining, Qinghai 810016, China; Qinghai Provincial Key Laboratory of Animal Ecological Genomics, Northwest Institute of Plateau Biology, Chinese Academy of Sciences, Qinghai 810008, China; University of Chinese Academy of Sciences, Beijing 100049, China

**Keywords:** environmental stress, hypobaric hypoxia, gut microbiota, microbe–microbe interplays, metagenomics, rats

## Abstract

In host–microbe interactions, host diet and environmental stress are key driving factors shaping the gut microbiota. Although previous studies have shown that hypoxia affects the structure and function of the gut microbiota in rodents, most have relied on 16S rRNA gene sequencing and lacked analysis of community assembly mechanisms, co-occurrence networks, and functional pathways. Here, we used metagenomic next-generation sequencing (mNGS) to examine the gut microbiota of rats exposed to hypobaric hypoxia (WH, simulated 6000 m altitude) compared to WL group (2100 m altitude). Hypoxia significantly altered β-diversity of gut microbiota, but did not affect its α-diversity. Community assembly was primarily governed by stochastic processes, with hypoxia stress reducing their impact. Microbial co-occurrence networks were dominated by positive correlations, although network resilience and stability declined under hypoxia. *Helicobacter* and *Eubacterium* were identified as high-abundance differentiating genera, and *Akkermansia muciniphila* was significantly enriched in WH group. Functional analysis revealed alterations in pathways related to protein synthesis and carbohydrate metabolism, suggesting that hypoxia may affect nutrient utilization by the host. Overall, these findings provide a comprehensive view of how hypoxic stress reshapes the gut microbiota of rats, offering new insights into microbial dynamics under environmental stress.

## Teaser

Hypobaric hypoxia disrupts gut microbiota stability and function in rats, potentially altering how the body uses nutrients.

## Introduction

Gut microbiota is a diverse community of microorganisms residing in the gut, including bacteria, fungi, viruses, and archaea (Portune et al. 2016). Among these, bacteria are the most abundant, with Bacillota (formerly Firmicutes) and Bacteroidota being the predominant groups in humans and rodents (Milani et al. 2020). Microbial community plays a crucial role in host health. Research indicates that the absence of gut microbiota during early life can impair gut sensory-motor functions and hinder neuronal development (Desbonnet et al. 2015, Heiss and Olofsson 2019) Additionally, gut microbiota significantly influences the production of various metabolites, including short-chain fatty acids (SCFAs), neurotransmitters, and amino acids. These metabolites affect gut barrier function and facilitate communication within the gut-brain axis through immune, endocrine, vagus nerve, and humoral pathways (Li et al. 2024a, Portune et al. 2016, Ma et al. 2022). Environmental stresses have been shown to disrupt gut microbiota, leading to an imbalance that exacerbates homeostasis disruption caused by stress (Montagnani et al. 2023). One such stressor is hypobaric hypoxia, which occurs in high-altitude environments, where reduced oxygen partial pressure results in insufficient oxygen supply to tissues (Maiti et al. 2006). This decrease in oxygen pressure inhibits diffusion from the alveoli into the bloodstream and tissues, causing in cellular hypoxia, the extent of which depends on the severity, duration, and type (Dunn et al. 2016, Li et al. 2021). Hypoxia disrupts the gut microbiota through three primary mechanisms: it damages the integrity of the mucosal barrier, increasing permeability and inflammation, which alters microbial composition and spatial distribution (Ma et al. 2023, Yan et al. 2024); it impairs digestive enzyme activity and smooth muscle peristalsis, reducing nutrient absorption and indirectly reshaping the microbiota by changing nutrient availability (Yang et al. 2021, Huang et al. 2025); and it modifies the epithelial microenvironment, including oxygen gradients, mucus thickness, and antimicrobial peptides, thereby altering the colonization niches for microbes (Li et al. 2022, Zhou et al. 2025).

Hypoxia stress significantly impacts the composition and function of the gut microbiota in animals (Ma et al. 2019). Following acute hypoxia exposure, the abundance of aerobic bacteria declines, while facultative anaerobic bacteria increases in the gut (Bai et al. 2022). In mammals, the ratio of Bacillota to Bacteroidota (Firmicutes/Bacteroidota, F/B) significantly decreases with prolonged hypoxia (Wang et al. 2021, Wang et al. 2022), although this trend is not significant during the early stages of hypoxic stress (Han et al. 2022). Hypoxia increases the abundance of opportunistic pathogen (mainly *Escherichia, Klebsiella, Enterococcus*, etc.), elevating the risk of bacterial translocation, and leading to extensive inflammatory responses (Singhal and Shah 2020). For instance, hypoxia enhances the proportion of Enterobacteriaceae and promotes the accumulation of virulence factors in the gut microbiota of rats (Ramos-Romero et al. 2020). Studies utilizing 16S RNA amplicon sequencing and PICRUSt2 prediction, indicate that hypoxic stress significantly alters the metabolic pathways of the gut microbiota in rodents (Bai et al. 2022). Both acute, chronic and intermittent hypoxia affect the composition and function of the gut microbiota in rats, with chronic stress having the most profound impact (Tian et al. 2018, Bai et al. 2022). Long-term environment stress and imbalance of the gut microbiota form a feedback loop, the foundation of which is the interaction between the host organism and the gut microbiota under the stress (Zhou et al. 2025).

Environmental stress not only affects the composition and function of the animal’s gut microbiota, but also influences interactions among microorganisms, including the community assembly mechanisms and co-occurrence networks (Lin et al. 2022). As the altitude increases, the assembly process of the gut microbiota community of plateau pika shifts from stochastic to deterministic processes, and the diversity and complexity of the microbial co-occurrence network have significantly decreased (Zhang et al. 2025). Several studies have reported the effects of hypoxia on the gut microbiota of rats (Tian et al. 2018, Ma et al. 2023). These studies primarily utilize 16S rRNA gene amplicon sequencing, focusing on changes in the α-diversity and β-diversity, the screening of differential microorganisms, and KEGG functional pathway prediction of the gut microbiota. However, with the development of Metagenomic Next-Generation Sequencing (mNGS), bacterial sequences can now be identified beyond the V3–V4 conserved regions of the 16S rRNA gene, allowing for species-level taxonomic annotation and functional pathway analysis at the GO and CAZy levels (Yen and Johnson 2021). Despite these advancements, the application of metagenomics to study the gut microbiota of rats has been limited at present, particularly regarding the analysis of important characteristics such as community assembly mechanisms and co-occurrence networks, which have been widely studied in small mammal (Lin et al. 2022, Mu et al. 2024, Li et al. 2025).

The rat was selected as the experimental model due to its well-characterized physiological response to hypoxia, its established utility in microbiome research for controlling confounding variables, and its suitability for generating sufficient microbial DNA to support deep metagenomic functional analyses (Tian et al. 2018, Bai et al. 2022, Han et al. 2022). Previous studies have not explored how the community structure, co-occurrence network, and function pathways of the gut microbiota in rats change under hypoxic stress. We constructed a microbial gene set of the rat gut microbiota through mNGS. And then we hypothesized that hypobaric hypoxia would: (i) drive community assembly toward deterministic processes (reducing stochastic processes), (ii) increase network connectivity while decreasing resilience, and (iii) promote the enrichment of function pathways related to carbohydrate and energy metabolism. To test these hypotheses, we integrated metagenomic profiling with assembly inference, co-occurrence networks, and multi-layer functional annotation. We analyzed these to reveal the comprehensive effects of hypobaric hypoxia on the gut microbiota of rats, and to provide background data for further research on the multi-disease co-occurrence mechanisms (gut–brain axis, gut–lung axis, etc.) under hypoxia.

## Materials and methods

### Animals and treatment

Adult male Wistar rats (*Rattus norvegicus*, 6∼8 weeks old; body mass ranges from 160∼210 g) were housed under SPF conditions. After a 2-week acclimation, using random digits table method, rats were randomly assigned by body mass stratification into an ambient-altitude control group maintained at 2100 m (WL, *N* = 20) and a hypobaric hypoxia group exposed to simulated 6000 m (WH, *N* = 20) in a hypobaric chamber (DYC-3000) for 28 days. An independent samples *t*-test was performed to compare body weight differences, confirming no baseline differences between groups. Husbandry (diet formulation, feeding time, cage-change schedule, and handling frequency) was kept identical between groups, and chamber environmental parameters (temperature, atmospheric pressure, oxygen pressure, oxygen concentration, and ventilation rate) were monitored and reported (Supplementary Table S1). After the 28 days of hypoxia, all individuals (WH & WL) were anesthetized by intraperitoneal injection of tribromoethanol (TBE, 2.5%, at a dose of 6 ml kg^−1^) and then euthanized. All animal experimental procedures were approved by the Animal Protection and Use Committee of the Northwest Institute of Plateau Biology, Chinese Academy of Sciences (NWIPB-2024–052). In accordance with *the 3R Principles* and the *ARRIVE 2.0 Guidelines*, every effort was made to minimize the number of animals used and their suffering.

### DNA isolation and library construction

The contents of the cecum of each rat were collected, rapidly frozen with liquid nitrogen, and then stored at −80°C in a refrigerator. To avoid selection bias, the gut contents samples of every three individuals were pooled into one sequencing sample, and there were 6 sequencing samples in each group (*n* = 6). Total DNA was isolated from sample using a QIAamp^®^ Fast DNA Stool Mini Kit (Qiagen, Hilden, Germany). DNA concentration and integrity were assessed by a NanoDrop2000 spectrophotometer (Thermo Fisher Scientific, Waltham, MA, USA) and agarose gel electrophoresis, respectively. All the samples’ DNA showed a main band, and the total amount met the requirements for library construction. DNA was fragmented by S220 Focused-ultrasonicators (Covaris, USA) and cleaned up by Agencourt AMPure XP beads (Beckman Coulter Co., USA). Then the libraries were constructed using TruSeq Nano DNA LT Sample Preparation Kit (Illumina, San Diego, CA, USA).

### Sequencing and quality control

The libraries were sequenced on BGI DNBSEQ-T7 platform and 150 bp paired-end reads were generated. Sequences in the FastQ file were trimmed and filtered using Trimmomatic (v 0.36). The post-filtered pair-end reads were aligned against the host genome using bowtie2 (v 2.2.9) and the aligned reads were discarded. Metagenome assembly was performed using MEGAHIT (v 1.1.2) after getting valid reads. Use gaps inside scaffold as breakpoint to interrupt the scaffold into new contigs, and these new contigs with length >500 bp were retained. ORF prediction of assembled scaffolds using Prodigal (v 2.6.3) was performed and translated into amino acid sequences. The non-redundant gene sets were built for all predicted genes using CDHIT (v 4.5.7). The clustering parameters were 95% identity and 90% coverage. The longest gene was selected as representative sequence of each gene set. Clean reads of each sample were aligned against the non-redundant gene set (95% identity) using bowtie2 (v 2.2.9), and the abundant information of the gene in the corresponding sample was counted.

The taxonomy of the species was obtained as a result of the corresponding taxonomy database of the NR library, and the abundance of the species was calculated using the corresponding abundance of the genes. In order to construct the abundance profile on the corresponding taxonomy level, abundance statistics were performed at each level of Domain, Kingdom, Phylum, Class, Order, Family, Genus, and Species. The gene set representative sequence (amino acid sequence) was annotated with NR, KEGG, and GO database with an e-value of 1e-5 using DIAMOND (v 0.9.7). The gene sets were compared with the CAZy database using the corresponding tool hmmscan (v 3.1) to obtain the information of the carbohydrate active enzyme corresponding to the gene and then calculated the carbohydrate activity using the sum of the gene abundances corresponding to the carbohydrate active enzyme abundance.

### Statistical analysis

Bacterial community composition was visualized using relative abundance histograms (phylum and genus levels) and Sankey diagrams. The α-diversity indices (Obs, Shannon, Simpson, Pielou, Chao1, ACE) and the β-diversity (based on Bray–Curtis distances) were calculated with the “vegan” package. Principal coordinate analysis (PCoA) was performed for ordination. Permutational multivariate analysis of variance (PERMANOVA, 999 permutations) was used to test for community differences, and homogeneity of multivariate dispersions (PERMDISP, 999 permutations) was assessed with betadisper (Oksanen et al. 2025). Further, β-diversity was decomposed into balanced variation and abundance gradients using the “betapart” package. (Baselga et al. 2012). Community assembly mechanisms were evaluated via the normalized and modified stochasticity ratios (NST, MST) using the “NST” package (Ning et al. 2019), and the neutral community model (NCM) was fitted with the “Hmisc” package (Harrell 2025).

In order to determine whether the intensity of hypoxia stress would lead to a change in community type, we selected the differential bacterial genera with the highest abundance ranking, and used the “DirichletMultinomial” and “magrittr” packages to establish a Dirichlet Multinomial Mixtures (DMM) model (Holmes et al. 2012, Bache and Wickham 2022). Between-group taxon abundances were compared using *t*–tests with Benjamini–Hochberg (BH) false discovery rate correction. Linear discriminant analysis effect size (LEfSe, LDA > 2, *P* < 0.05) was performed across all taxonomic levels with the “microeco” package (Liu et al. 2021). And the LEfSe results were cross-validated with ANCOM-BC (log_2_ FC, BH–adjusted q–values) with the “ANCOMBC” package (Lin and Peddada 2020, Lin and Peddada 2024). Functional differences were analyzed using KEGG, GO, and CAZy annotations. Differential pathways were identified by *t*–tests and LEfSe (LDA > 2, *P* < 0.05).

Co–occurrence network analysis was conducted using “microeco” and “WGCNA” packages based on Random Matrix Theory (Langfelder and Horvath 2012). Spearman correlations, with the filtering threshold set at |r| > 0.80 and *P* < 0.05, were screened. The networks were constructed with “igraph,” and visualized in Gephi (v 0.10.1). The Fruchterman Reingold layout mode was used, with species colors representing the taxonomic label at the phylum, point size representing abundance, and line width representing the strength of correlation (Csardi and Nepusz 2006). Topological properties (nodes, edges, average degree, modularity, etc.) and cohesion (negative cohesion, positive cohesion, the ratio) were computed. All analyses were performed in R 4.3.2, and figures were generated with “ggplot2”, “ggsci,” and “ggpubr.”

## Results

### Changes in the structure of the gut microbiota

A total of 10 232 species in Bacteria, accounting for 98.538% ± 0.212%, 702 species in Viruses, accounting for 1.413% ± 0.210%, 205 species in Archaea, accounting for 0.041% ± 0.004%, and 640 species in Fungi, accounting for 0.008% ± 0.019%, were detected in the gut microbiota of rats. Therefore, we mainly focused on the structural changes of the bacterial community. At the phylum level, Bacillota had the highest abundance in WH (56.54% ± 7.51%), followed by Bacteroidota (36.59% ± 6.96%) and the Campylobacterota (1.32% ± 0.89%). In WL, Bacillota also had the highest abundance (56.85% ± 4.04%), followed by Bacteroidota (36.57% ± 4.36%) and the Deferribacterota (0.99% ± 0.36%). At the genus level, *Prevotella* had the highest abundance in both WH and WL (WH: 10.68% ± 5.46%; WL: 12.95% ± 3.47%), followed by *Bacteroides* (WH: 4.57% ± 0.88%; WL: 3.77% ± 0.41%) and *Oscillospira* (WH: 2.73% ± 1.58%; WL: 2.38% ± 0.26%). Except for *Lawsonibacter* and *Phocaeicola*, there were no significant differences in the abundance of other dominant phyla and genera between the two groups (Supplementary Table S2). The Sankey diagram indicated that the top 30 bacterial genera in the rat gut microbiome mainly belong to Bacteroidota, mostly being Gram-negative anaerobic or facultative anaerobic bacteria (Supplementary Fig. S1). The α-diversity indices of the rat bacterial community, including Obs, Shannon, Simpson, Pielou, Chao 1, and ACE, showed no significant differences (Supplementary Table S3).

**Figure 1:**
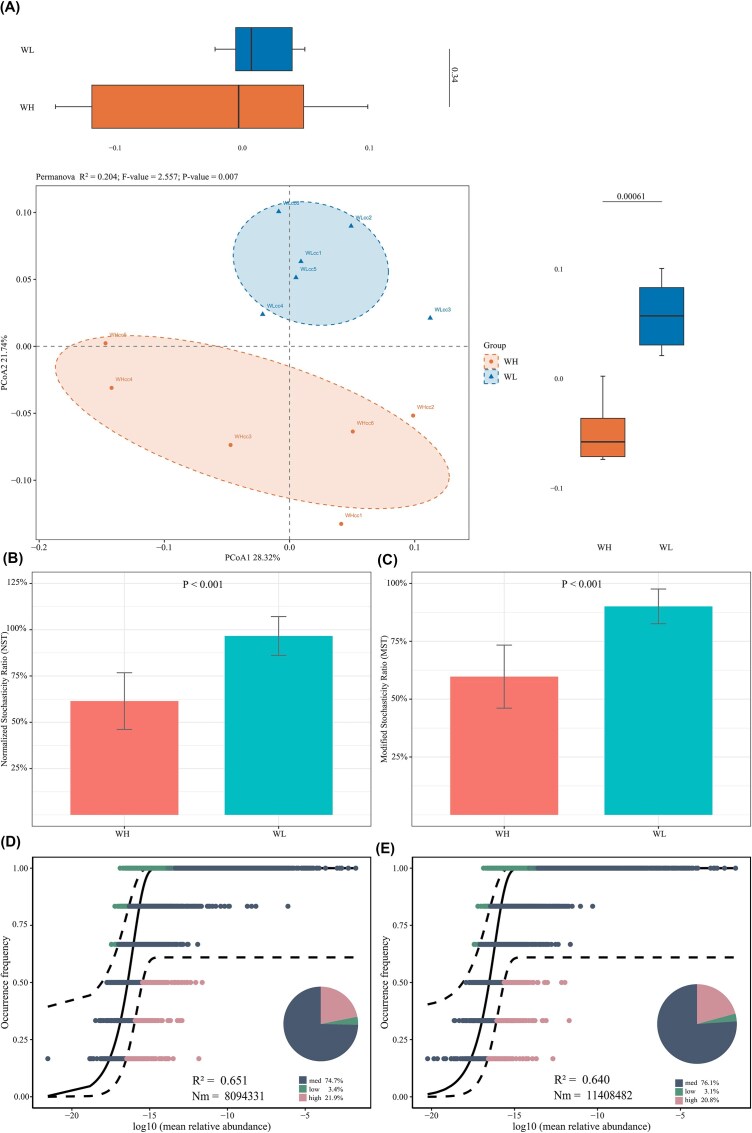
β-diversity of gut microbiota and community construction mechanism in rats. (A) PCoA and PERMANOVA results; (B) NST value of each group; (C) MST value of each group; (D) NCM result of WH; (E) NCM result of WL.

The β-diversity was clustered by Bray–Curtis distance. PCoA1 explained 28.32% of the variation, and PCoA2 explained 21.74% of the variation. PERMANOVA results revealed significant differences in bacterial community species composition between WH and WL (*F* = 2.557, *P* = 0.007), and PERMDISP indicated that within-group dispersion was significantly higher in WH than in WL (*F* = 7.981, *P* = 0.026). The results of the β-diversity decomposition indicated that balanced variation was dominant (balanced variation/total variation = 55.49%/58.20%), and the structural changes in the community composition were the main factors driving the significant PERMANOVA. Both groups had NST and MST >0.5, suggesting that the bacterial community construction was mainly dominated by stochastic processes. However, the NST and MST of WH were significantly lower than those of WL (*P* < 0.001), suggesting that the contribution proportion of the stochastic process was significantly reduced. Bacterial communities of both groups fitted relatively with NCM (R^2^ > 0.60). However, in the case of similar metacommunity scales (N_WH_ = 25 480 539.899; N_WL_ = 24 737 736.540), the diffusion migration amount of WH was lower (Nm_WH_ = 8 094 331; Nm_WL_ = 11 408 482), suggesting that the community assembly was affected by the limitations of migration (Fig. 1).

### Differential analysis of the gut microbiota

**Figure 2:**
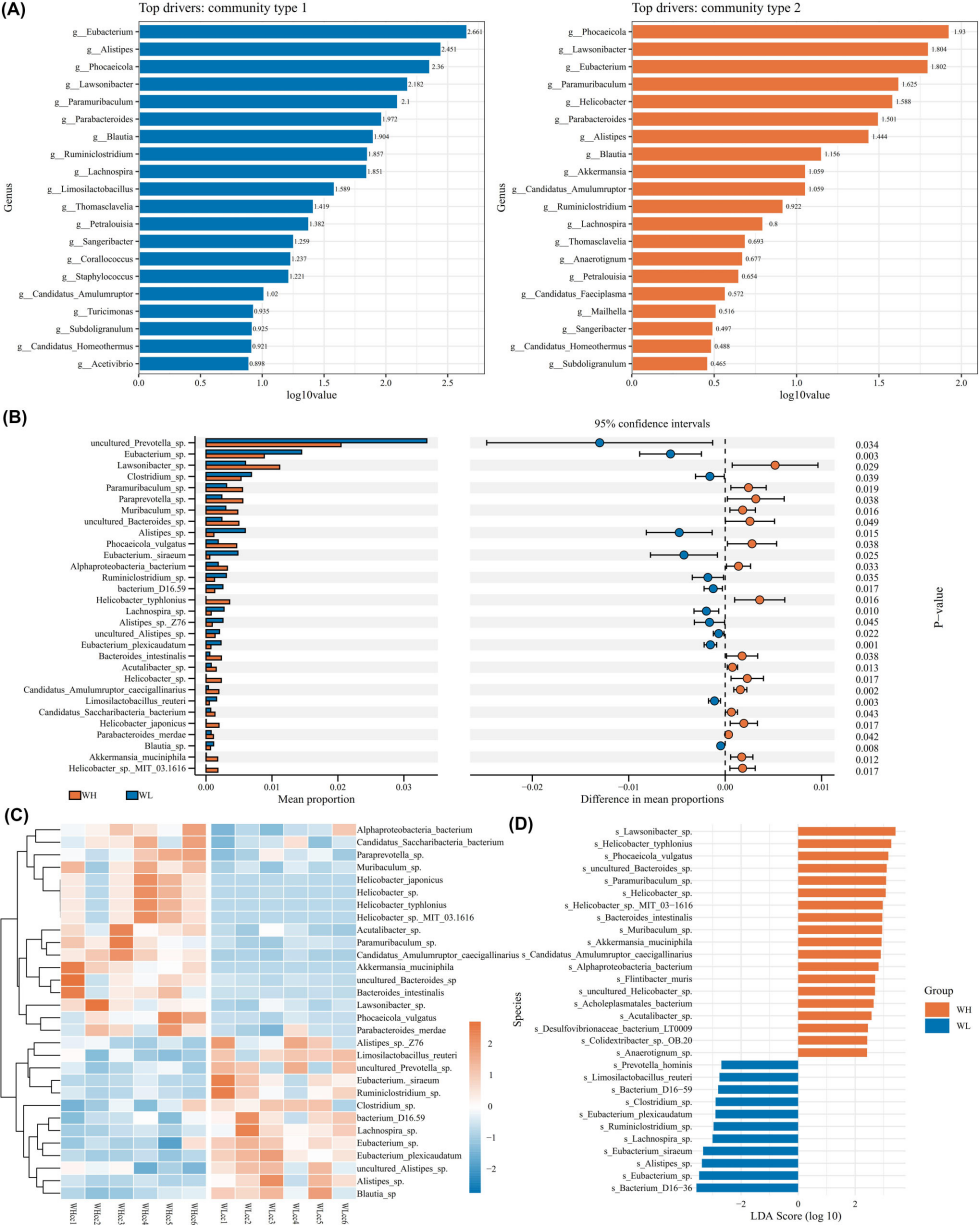
DMM typing and differential classification units of gut microbiota in rats. (A) The DMM typing results of the bacterial community; (B) Stamp plot of high-abundance different species; (C) Clustering heatmap of high-abundance different species (D) LEfSe score of high-abundance different species.

To evaluate the contribution of bacterial genera to community typing, the top 200 most abundant differential genera were selected to construct a DMM model. The optimal model fit was achieved when the number of Dirichlet components was two, indicating that the rat gut microbiota could be partitioned into two distinct community types. For type 1, the top five contributing genera were *Eubacterium, Alistipes, Phocaeicola, Lawsonibacter*, and *Paramuribaculum*, in descending order of contribution. For type 2, the top five were *Phocaeicola, Lawsonibacter, Eubacterium, Paramuribaculum*, and *Helicobacter*. With ANCOM-BC method, a total of 3232 differentially abundant bacterial species (*q* < 0.05) were identified. Among them, 481 species were significantly enriched in the WH group (*q* < 0.05, log_2_ FC > 1), 1597 species were significantly enriched in the WL group (*q* < 0.05, log_2_ FC < −1). Differential species between groups were further identified using ANCOM-BC and LEfSe, and the results revealed that several *Helicobacter* species, including *Helicobacter typhlonius, Helicobacter* sp., and *Helicobacter* sp*. MIT-03–1616*, were significantly enriched in the WH group (LEfSe: LDA > 2, *P* < 0.05; ANCOM-BC: log₂ FC > 1, *q* < 0.05). Additionally, *Akkermansia muciniphila, Limosilactobacillus reuteri*, and *Prevotella hominis* also exhibited significant differences between groups (LEfSe: LDA > 2, *P* < 0.05; ANCOM-BC: |log₂ FC| > 1, *q* < 0.05; Fig. 2 & Supplementary Table S4).

**Figure 3:**
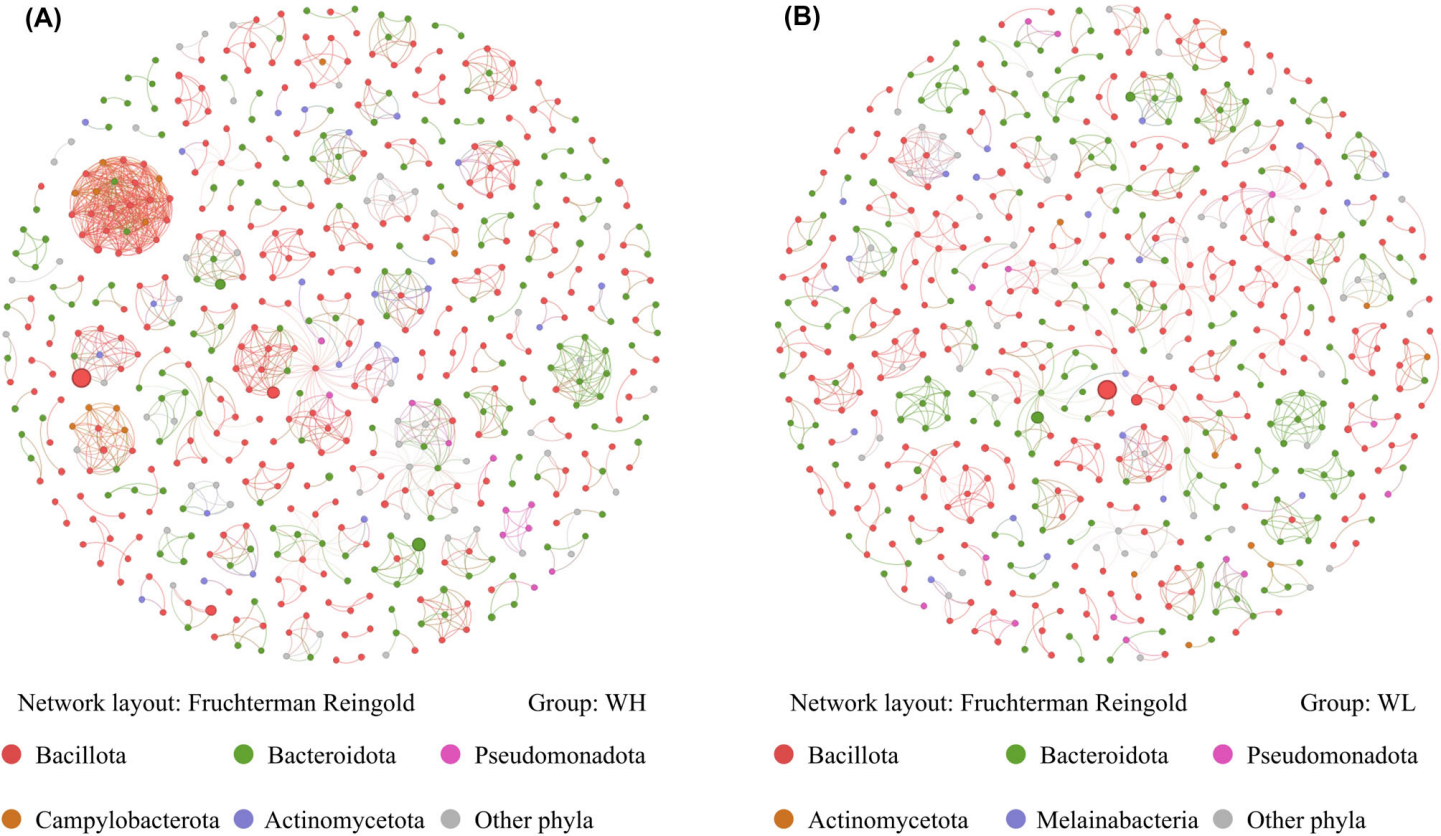
Co-occurrence network of gut microbiota in different groups of rats. (A) The network of WH; (B) The network of WL. The color of nodes represented the phylum; the color of edges represented the positive and negative correlation; the width of edges represented the strength of the correlation.

We constructed a microbial co-occurrence network to explore the interactions of the intestinal microbiota. In WH, 562 nodes and 1278 edges were selected, and in WL, the figures were 565 and 930 (Fig. 3). In both networks, most nodes belonged to Bacillota (WH: 51.07%, WL: 50.80%) and Bacteroidota (WH: 31.67%, WL: 33.45%), and most edges showed positive correlations between species (WH: 59.31%, WL: 66.34%). The topological properties of the two networks differed markedly. The average degree, clustering coefficient, heterogeneity, and centralization of the WH network were 38.15%, 23.79%, 54.44%, and 64.52% higher than those of the WL network, respectively. The average path length of the WH network was 31.50% lower than that of the WL network. These indicated that the network structure in WH was closely connected and had a strong community structure, and there were important nodes with high connectivity in the network. The modularity of both communities was >0.90, suggesting that there were obvious modules in the network, with connections mainly existing within the modules and fewer between them. The negative cohesion of the WH network was significantly more negative than that of the WL network (*F* = 1.804, *P* = 0.002), the positive cohesion was significantly more positive than that of the WL network (*F* = 1.330, *P* < 0.001), and NC: PC was significantly lower (*F* = 2.971, *P* < 0.001), suggesting that the stability of the microbial network was affected under low oxygen stress, and the resilience decreased (Table 1).

**Table 1:** Trait index and cohesion of co-occurrence network of gut microbiota in rats

Index	WH	WL	*F*	*P*
Vertex	562	565		
Edge	1278	930		
Average degree	4.548	3.292		
Average path length	1.362	1.982		
Network diameter	3	6		
Clustering coefficient	0.921	0.744		
Heterogeneity	1.078	0.698		
Centralization	0.051	0.031		
Modularity	0.930	0.976		
Negative Cohesion	−0.381 ± 0.004	−0.372 ± 0.003	1.804	0.002
Positive Cohesion	0.443 ± 0.006	0.415 ± 0.003	1.330	<0.001
Negative: Positive Cohesion	0.860 ± 0.135	0.897 ± 0.005	2.971	<0.001

### Changes in the function of the gut microbiota

**Figure 4:**
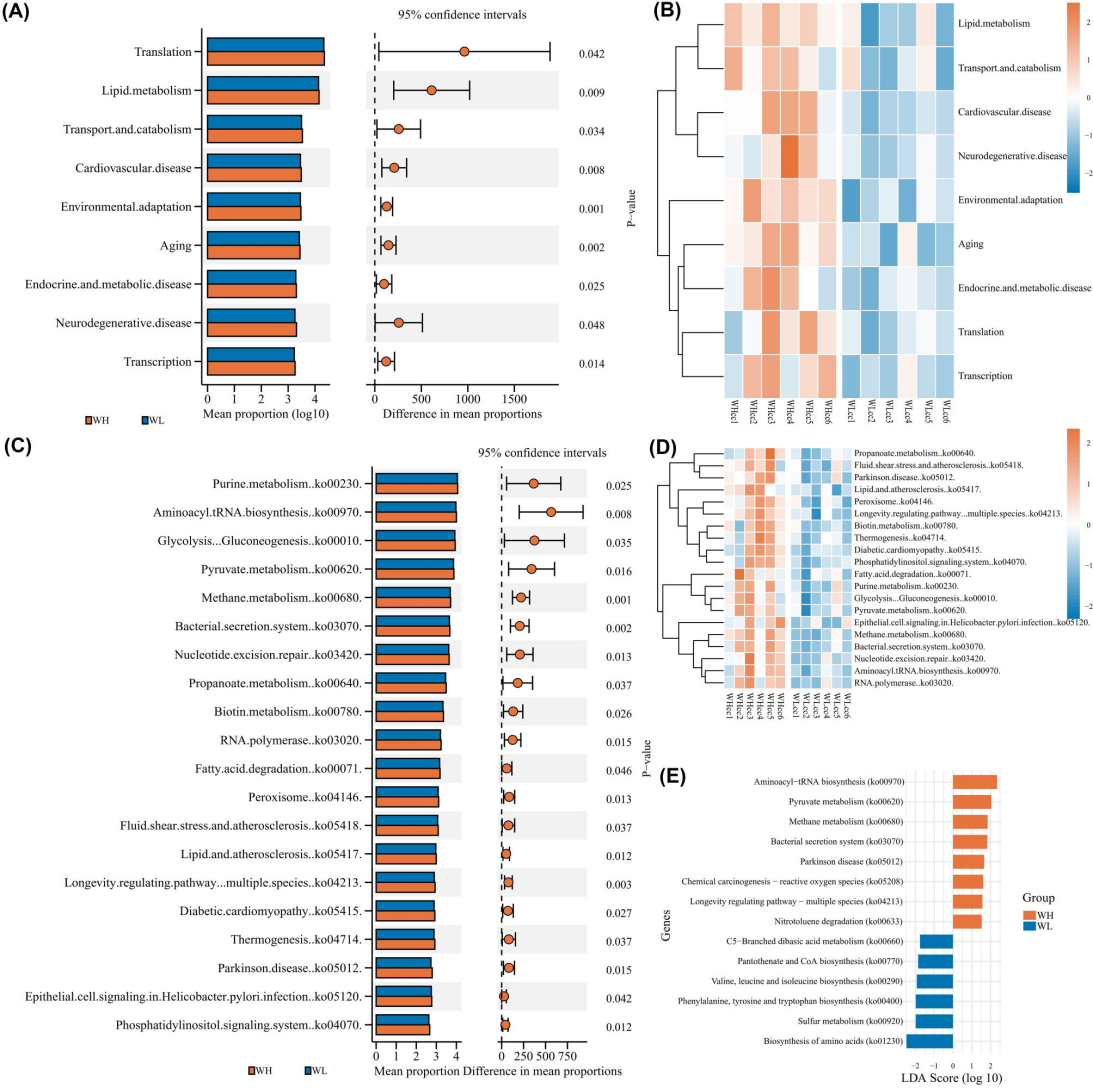
Enrichment of KEGG pathways of the gut microbiota in rats. (A) Stamp plot of high-expression differential pathways in KEGG Level2; (B) Clustering heatmap of high-expression differential pathways in KEGG Level2; (C) Stamp plot of high-expression differential pathways in KEGG Level3; (D) Clustering heatmap of high-expression differential pathways in KEGG Level3; (E) LEfSe score of high-expression differential pathways in KEGG Level3.

The KEGG functional annotations were conducted for the gut microbiota of rats, and a total of 448 KEGG Level3 pathways were annotated, belonging to 48 KEGG Level2 pathways, respectively involving cellular processes, environmental information processing, genetic information processing, human disease, metabolism, and organismal systems (Supplementary Fig. S2). Based on the KO database, the homologous genes of the rat gut microbiota showed significant down-regulation for *K07497, hsdS, hsdR, phoB1, phoP*, etc. (*P* < 0.01), and significant up-regulation for *LARS, lcns, VARS, vals, ATP2C*, etc. (*P* < 0.01). The functional profiles of the gut microbiota in the WH group were significantly enriched in Level2 pathways, such as Translation, Lipid metabolism, Transport and catabolism, etc. (*P* < 0.05). KEGG Level3 functional pathways, including amino acid biosynthesis (ko01230), aminoacyl-tRNA biosynthesis (ko00970), and pyruvate metabolism (ko00620), were significantly enriched in the WH group (LDA > 2, *P* < 0.05, Fig. 4).

**Figure 5:**
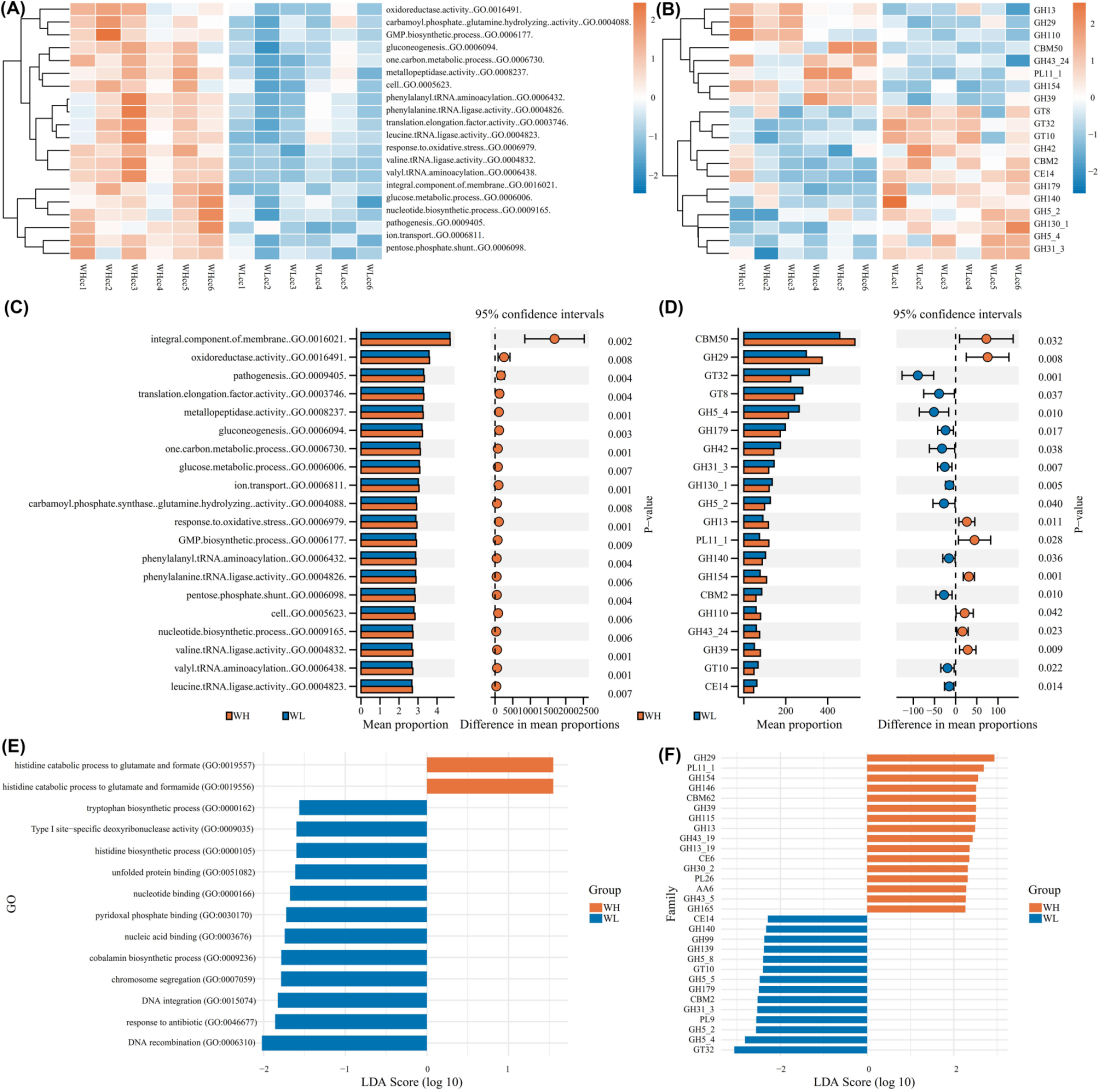
Enrichment of GO pathways and CAZy library in the gut microbiota of rats. (A, B) Clustering heatmap of high-expression differential pathways in GO and CAZy; (C, D) Stamp plot of high-expression differential pathways in GO and CAZy; (E, F) LEfSe score of high-expression differential pathways in GO and CAZy.

GO functional annotation was performed, covering a total of 10 453 pathways, which were divided into three parts: cellular component (CC), molecular function (MF), and biological process (BP) (Fig. 5). Pathways such as membrane components (GO.0016021), redox enzyme activity (GO.0016491), and pathogen (GO.0009405) were significantly enriched in the WH group (*P* < 0.05). CAZy library was annotated with 479 carbohydrate enzyme families (CAZy family), belonging to 6 classes (CAZy class), including glycoside hydrolases (GHs), glycosyltransferases (GTs), polysaccharide lyases (PLs), glycerol esterases (CEs), and auxiliary active redox enzymes (AAs), as well as a domain for carbohydrate binding (CBMs). The differentially expressed enzymes were mainly distributed in the three major classes: GHs, GTs, and PLs. GH39, GH13, PL11_1 showed significantly upregulated expression, while GT32, GH5_4, GH179 showed significantly downregulated expression (LDA > 2, *P* < 0.05).

## Discussion

### Hypoxia affected the bacteria abundance and community assembly mechanism

Cross-species studies have shown that the host species played a dominant role in shaping the microbiota of small mammals, while intra-species studies emphasized that host diet and environmental factors were important factors in shaping the gut microbiota (Knowles et al. 2019, Zhao et al. 2022). The mammalian intestine was in a highly perfused state and was highly sensitive to hypoxia and ischemia. Changes in the intestinal mucosal state affected the colonization of gut microbiota (Singhal and Shah 2020). After hypoxia exposure, the gut microbiota of rats still mainly consisted of the Bacillota and Bacteroidota phyla, but the abundance of Proteobacteria in WH increased. Proteobacteria contained many pathogenic or opportunistic pathogenic bacteria, such as *Escherichia coli, Salmonella, Helicobacter pylori*, and so on. The increase in their abundance would increase the risk and susceptibility of bacterial exposure in the gut (Bai et al. 2022). There was no significant difference in the α-diversity of the rat gut microbiota among different treatments, which was consistent with the results of other experimental studies, suggesting that the richness, evenness, and rare species richness of the community remained balanced under hypoxia stress (Zhang et al. 2018, Lin et al. 2022). In our data, hypoxia primarily drove both a shift in community composition and a change in within-group dispersion, as supported by PERMANOVA and PERMDISP results. The results of the decomposition of β-diversity indicate that balanced variation in abundance was dominant (55.49%), and the structural change of the core composition of the community (position effect) was the main factor driving the significant PERMANOVA. Field studies have shown that after exposure to low oxygen stress, the β-diversity of the microbial community was mainly composed of turnover components, indicating that the environment has selected specific adaptive microbial communities (Wu et al. 2022). Environment stress can alter both the centroid and dispersion of microbial communities, which were also observed in the cold stress experiment (Lv et al. 2024, Mu et al. 2024).

In host–microbe interaction system, host hereditary features, microbial interactions, and environmental factors were regarded as the three major factors influencing the assembly of the microbiota. Habitat filtering mediated by the environment was considered to play a more significant role than host genetic background (Rothschild et al. 2018). Our results indicated that under hypoxic conditions, the assembly of the gut microbiota in rats was dominated by the stochastic process, but hypoxic stress significantly reduced the contribution of the stochastic process by hindering migration and diffusion. The construction process of the gut microbiota in rats conformed to the NCM, suggesting that the environment remained a homogeneous environment, and the persistence of species with lower competitive ability in the homogeneous environment was based on the diffusion limitation of species with high diffusion ability (Logue et al. 2011). Hypoxia limited the energy supply for the synthesis and assembly of flagella in aerobic bacteria, downregulated the expression of genes related to flagellum assembly in specific bacteria (Matilla and Krell 2018, Grzymajlo et al. 2023). Hypoxia also altered the gut metabolism, affecting the availability of key chemical attractants such as bile acids and SCFAs, thereby influencing the directional migration of bacteria (Jyoti and Dey 2025, Yang et al. 2025). The low homogenizing dispersal limited the exchange of species between communities, which reduced stochastic processes into the community (Evans et al. 2017, Chavez et al. 2025).

### Hypoxia affected the community type and the stability of co-occurrence network

Microbial communities from different environments, especially host-related oral microbial communities and intestinal microbial communities, could be classified into different types based on their microbial composition (Arumugam et al. 2011, Ding and Schloss 2014). The DMM classification results showed that the gut microbiota of rats was divided into two types, the dominant bacterial groups in the rat intestinal microbiota remained consistent and there were no structural subtypes (Gonze et al. 2017). Structural subtypes were observed in the gut microbiota of plateau pikas and toad-headed agama in hypoxia, this suggests that the effect of hypoxia on the remodeling of the community type in rats was limited, and the observed compositional changes should be more considered as the variation of specific bacterial abundance (Yu et al. 2022, Du et al. 2024). The high-contributing genera of rat gut typing mainly utilized carbohydrates and proteins, producing SCFAs such as acetic acid, propionic acid, and butyric acid, as well as their derivative salts. In WH, the abundance of *Phocaeicola, Lawsonibacter*, and *Helicobacter* increased, and their lipid metabolism ability might be correspondingly enhanced, and the risk of pathogenic exposure also increased (Ma et al. 2023).

Uncultured bacteria and Candidatus accounted for >80% of the gut microbial community (Pu et al. 2023). It was difficult to infer the direct interactions among species, so it was necessary to explore the interspecies relationships in the complex microbial community through co-occurrence network analysis (Ona et al. 2025). The interspecies relationships in rats were mainly positive correlations, suggesting that species mainly have symbiotic and reciprocal relationships, and the interaction network had a high degree of functional redundancy (Hernandez et al. 2021, Wu et al. 2023). The edge, average degree, heterogeneity, and centralization index of the co-occurrence network in WH were all higher than those in the WL group, while the modularity degree was slightly lower than that in WL. This suggested that the interactions among bacteria under hypoxic stress were more closely and complex, and there might be important nodes that maintain the stability of the network (Yang et al. 2023). The results of network cohesion showed that the positive and negative cohesion of the network in WH had significantly increased, and NC:PC had significantly decreased. These indicated that hypoxia has increased the interaction relationships among microflora in WH, but the species interaction still mainly manifested as collaborative relationships, such as sharing of nutrient resources and coexistence in the same habitat, and the anti-stress stability of the network has decreased. Hypoxia was a high-stress factor for the gut microbiota of rats (Herren and McMahon 2017, Freilich et al. 2018).

### Hypoxia led to differences in the abundance of gut microbiota

The role of a single bacterial species in the gut was complex, so we needed to pay attention to the common functional traits of the bacterial species belonging to the same bacterial genus (Taylor and Colgan 2017). In the gut microbiota of rats, the abundances of *Prevotella* sp., *Eubacteria* sp., and *Clostridium* sp. bacterial species all significantly decreased in WH, which was similar to the results of other studies conducted under similar conditions (Li et al. 2022, Wang et al. 2023). These bacterial species belonged to the bacterial genera that mainly produced butyrate and were decomposers of plant polysaccharides (Suzuki et al. 2019). Their colonization in the mucus layer increased the bioavailability of butyrate for intestinal epithelial cells, yet a few of them were pathogenic bacteria (Singh et al. 2022). Under conditions where the available carbohydrate content decreased, the intake ratio of protein and fat increased, or bile acid and lipid metabolism was abnormal, the abundances of *Prevotella* and *Eubacteria* could be observed to decrease, which was not conducive to the degradation of plant cellulose (Tett et al. 2021). The decrease in the abundance of these SCFAs-producing bacteria was related to the loss of intestinal wall integrity and excessive immune response, which led to inflammatory bowel disease or visceral allergy (Akhtar et al. 2021, Ma et al. 2022).

The abundances of *Akkermansia muciniphila* increased significantly in WH, which was similar to the results obtained in studies conducted in mice (Yan et al. 2024). *Akkermansia muciniphila* was associated with intestinal inflammation, but its role in promoting or inhibiting inflammation remained uncertain and debatable (Lei et al. 2023). *Akkermansia* was a sensitive bacterial population in response to hypoxia, and the change of its abundance was related to the condition of the mucus layer (Bai et al. 2022, Mishra and Bakshi 2023). Under the chronic hypoxia stress at 2000 m and 5000 m altitudes, the abundance of *Akkermansia* showed a significant increase, while under the stress at 7620 m, the abundance significantly decreases, which indicated that the abundance variation was related to the degree of stress (Khanna et al. 2019, Huang et al. 2021, Bai et al. 2022). The increase in *Akkermansia* induced by hypoxia was related to the increase in SCFAs production and the enhancement of exercise endurance (Huang et al. 2021). While, other studies suggest that increased abundance of *Akkermansia muciniphila* may exacerbate the inflammatory response by depleting the intestinal mucosal layer (Ganesh et al. 2013, Hakansson et al. 2015). Our results have confirmed the hypoxia sensitivity of *Akkermansia*, but the specific effects of this were yet to be verified through subsequent fecal microbiota transplantation experiments.

### Hypoxia led to differences in microbial functional pathways

The KEGG and GO functional pathways enriched in the gut microbiota of rats were mainly related to the synthesis of biological macromolecules and substance metabolism. Under hypoxic stress, the expression of genes related to transcription and translation of gut microbiota was upregulated, and ko homologous genes included *LARS* encoding cytoplasmic leucyl-tRNA synthetase and *VARS* encoding cytoplasmic valyl-tRNA synthetase. The gut microbiota regulated the availability of host amino acids through multiple mechanisms and the regulatory microorganisms and their metabolic genes were largely difficult to determine, so their synthesis pathways and fluxes were not yet completely clear (Li et al. 2024a, Portune et al. 2016). The KEGG pathway enrichment results, including amino acid biosynthesis (ko01230) and aminoacyl-tRNA biosynthesis (ko00970), indicated that the expression of tRNA synthetase encoding genes of the microbiota in WH was upregulated, which was consistent with the result that the tRNA ligase activity of the microorganisms enriched in the GO pathway was upregulated. These indicated that hypoxia affected the ability of microorganisms to synthesize bacterial proteins, which might lead to an increase in the concentration of intracellular virulence proteins in WH (Weitzel et al. 2020, Grob et al. 2022). The pathogenicity pathway (GO.0009405) was also significantly enriched in WH, which was related to the increase in the abundance of pathogenic bacteria, such as *Escherichia coli* and *Helicobacter pylori* within the group, increasing the risk of bacterial translocation and combined infection with pathogenic bacteria (Wang et al. 2022).

Hypoxia affected the metabolic capacity of the gut microbiota in rats. KEGG pathways such as purine metabolism (ko00230), pyruvate metabolism (ko00620), and propionate metabolism (ko00640) were significantly enriched in WH, suggesting that the utilization rate of nutrients by the gut microbiota was affected (Li et al. 2024b). The results of GO functional pathway enrichment showed that genes related to gluconeogenesis (GO.0006094) and glucose metabolism process (GO.0006006) were significantly enriched in WH, suggesting that the regulation of glucose metabolism by the gut microbiota changed. Undigested dietary polysaccharides were the main carbon source for the gut microbiota. After polysaccharides were decomposed into oligosaccharides by GHs outside the bacterial cells and transported into the cells by corresponding transport proteins for further degradation into pyruvate, and they were synthesized into glucose through gluconeogenesis (Wardman et al. 2022). Such processes were relatively common in the Bacteroidota and provide a basis for cross-feeding among different bacterial species (Mao et al. 2007). The CAZy enrichment results indicated that the expression levels of GHs such as GH29, GH39, and GH13 changed, which might be related to the changes in the carbohydrate cross-feeding network and ultimately manifested as the complexity of the interaction network in WH (Zeng et al. 2022, Clegg and Gross 2025).

Our research systematically analyzed the effects of chronic hypoxia stress on the composition, structure, and function, enriching the understanding of the community assembly and co-occurrence networks of the gut microbiota in rats. Additionally, our results indicated that *Akkermansia* was sensitive to hypoxic stress, but the specific mechanism of its action remains unclear. Although pooling samples have been widely used in experimental design, further research at the individual level will be warranted (Bai et al. 2022, Novoa et al. 2023). This could involve combining FMT experiments with Metatranscriptomics to better assess the general applicability of our findings and to explore the mechanisms of specific bacterial genera in greater depth.

## Conclusion

Our research indicated that the gut microbiota of rats exhibited a series of changes in response to hypoxia stress. Extreme hypoxia significantly affected the β-diversity of the gut microbiota, and reduced the contribution of stochastic processes in the community assembly mechanism. Hypoxia diminished the stability and resilience of the co-occurrence network, suggesting that the interactions within rats gut microbiota became more vulnerable under stress conditions. We observed a decrease in the abundance of butyrate-producing bacteria, such as *Prevotella* and *Eubacterium*, which were typically associated with the production of SCFAs. And there was an increase in the abundance of opportunistic pathogenic bacteria, including *Helicobacter* and *Escherichia*. The abundance of *Akkermansia* increased, demonstrating sensitivity to hypoxia and requiring further validation of its mechanism. Hypoxia also affected the functional profiles of the gut microbiota, enhancing protein synthesis and nutrient metabolism in the gut microbiota of rats. The structural and functional changes of the gut microbiota caused by hypoxic stress would affect the intestinal morphology and function of the host. Rats have always been excellent experimental animals for intervention experiments (fecal microbiota transplantation, drug treatment, etc.). This study, by combining mNGS sequencing technologies and statistical models, has revealed the comprehensive impacts of hypoxic stress on the gut microbiota of rats, which will be helpful for the subsequent conduct of comparative physiological or pharmacological experiments.

## Supplementary Material

fiag039_Supplemental_File

## Data Availability

The raw sequence data reported in this paper have been deposited in the Genome Sequence Archive (Genomics, Proteomics & Bioinformatics 2025) in National Genomics Data Center (Nucleic Acids Res 2025), China National Center for Bioinformation/Beijing Institute of Genomics, Chinese Academy of Sciences (GSA: CRA038720) that are publicly accessible at https://ngdc.cncb.ac.cn/gsa.
