# Supplementary material for: Hypobaric hypoxia affects gut microbiota of rats through affected community assembly, reduced network resilience, and metabolic reprogramming
Source: FEMS Microbiol Ecol. 2026 Apr 16;102(5):fiag039. doi: 10.1093/femsec/fiag039 (PMC13131234; doi:10.1093/femsec/fiag039)
Supplement: fiag039_Supplemental_File [file fiag039_supplemental_file.docx]

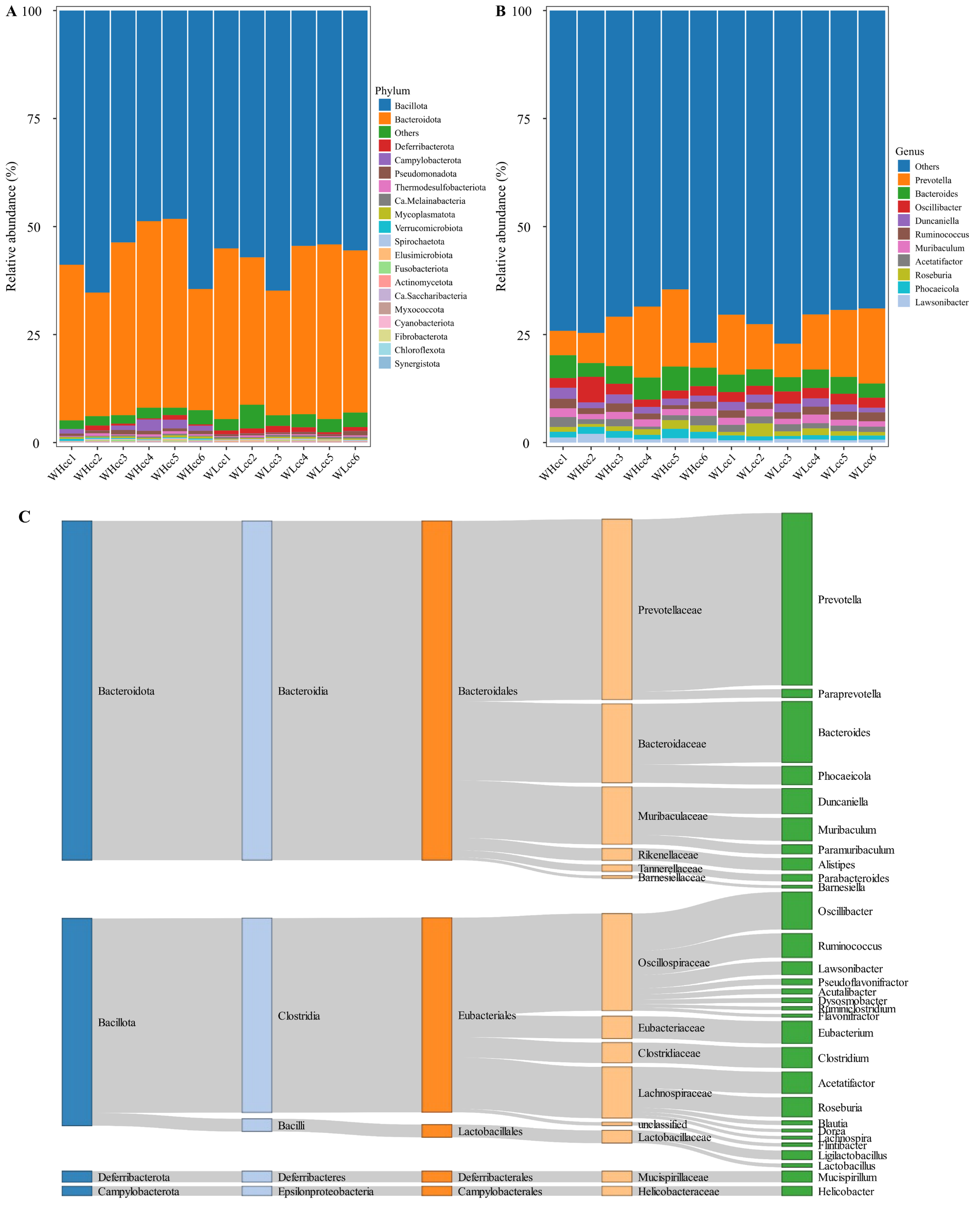


**Figure S1** Distribution of gut microbiota phylum and genus in rats. (A) Dominant phylum and their relative abundances; (B) Dominant genus and their relative abundances; (C) Dominant genus taxonomic status.


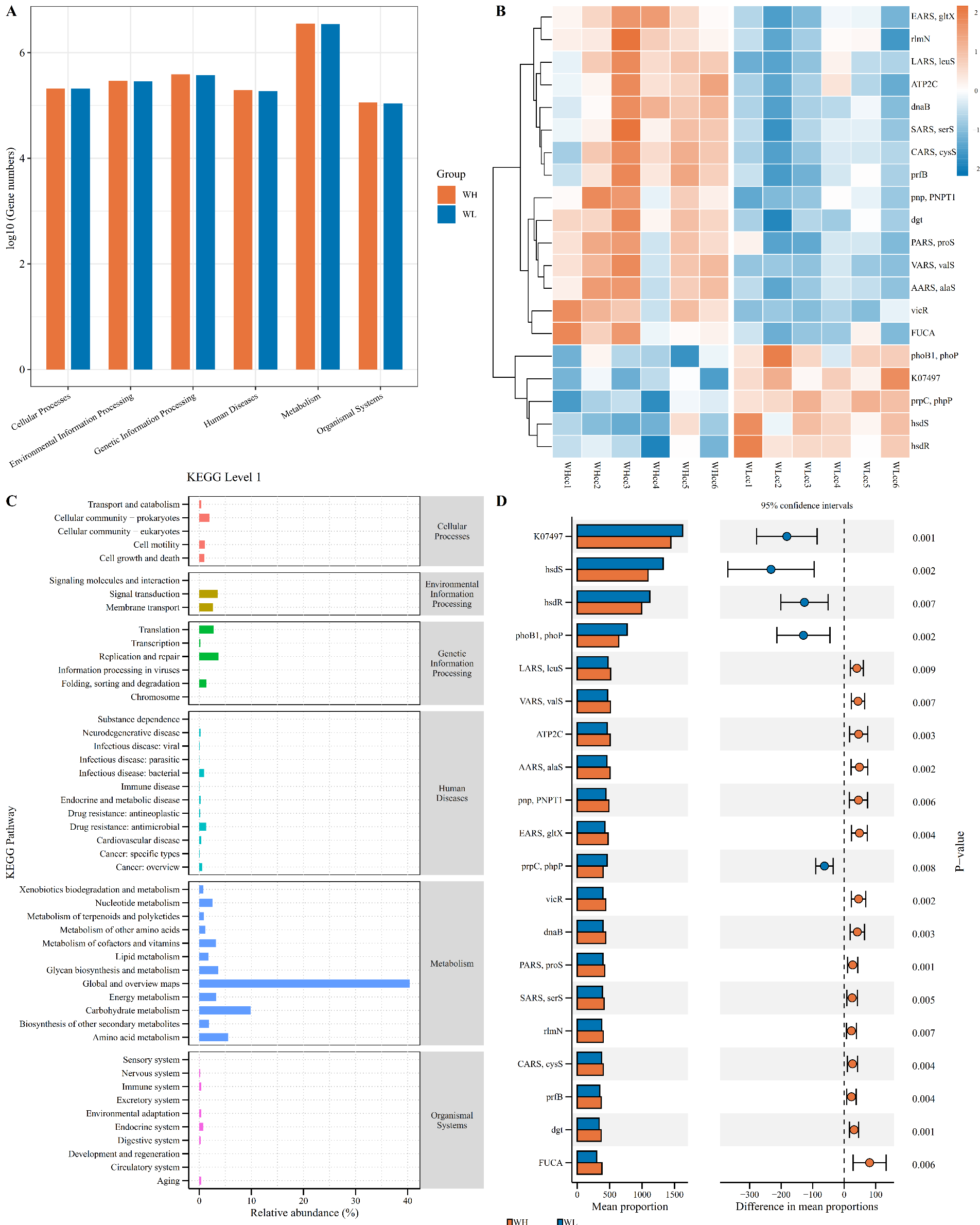


**Figure S2** Functional annotation of KEGG of the gut microbiota in rats. (A) The number of genes annotated to KEGG level 1; (B) Clustering heatmap of high-abundance different genes in KO; (C) The distribution of KEGG level 2 pathways at the level 1; (D) LEfSe score of high-abundance different homologous gene.

**Table S1 Hypoxic treatment conditions**

| Group | Temperature ℃ | Atmospheric Pressure kPa | Oxygen Pressure kPa | Oxygen Concentration % | Ventilation Rate m^3^·h^-1^ |
| --- | --- | --- | --- | --- | --- |
| WH | 20 ± 2 | 45.97 ± 0.50 | 8.96 ± 0.25 | 19.50 ± 0.20 | 120 |
| WL | 20 ± 2 | 77.79 | 15.16 | 19.49 | 120 |

**Table S2 The t-test results of the high-abundance phylum and genus of bacteria**

| Taxa | Name | WH abundance % | WL abundance % | *P* |
| --- | --- | --- | --- | --- |
| Phylum | Bacillota | 55.77 ± 7.46 | 55.97 ± 4.05 | 0.955 |
|  | Bacteroidota | 36.09 ± 6.83 | 35.99 ± 4.27 | 0.977 |
|  | Campylobacterota | 1.09 (1.03 - 1.76) | 0.04 (0.04 - 0.04) | 0.055 |
|  | Deferribacterota | 0.53 ± 0.46 | 0.97 ± 0.35 | 0.091 |
|  | Pseudomonadota | 0.74 ± 0.16 | 0.50 ± 0.24 | 0.063 |
| Genus | *Acetatifactor* | 1.49 ± 0.66 | 1.42 ± 0.21 | 0.810 |
|  | *Bacteroides* | 4.52 ± 0.88 | 3.72 ± 0.39 | 0.067 |
|  | *Duncaniella* | 1.71 ± 0.47 | 1.72 ± 0.33 | 0.976 |
|  | *Lawsonibacter* | 1.05 (0.97 - 1.15) | 0.60 (0.53 - 0.69) | 0.006 |
|  | *Muribaculum* | 1.58 ± 0.27 | 1.50 ± 0.29 | 0.641 |
|  | *Oscillibacter* | 2.20 (1.94 - 2.39) | 2.32 (2.25 - 2.41) | 0.423 |
|  | *Phocaeicola* | 1.52 ± 0.36 | 0.94 ± 0.14 | 0.005 |
|  | *Prevotella* | 10.53 ± 5.37 | 12.75 ± 3.43 | 0.414 |
|  | *Roseburia* | 1.17 (1.08 - 1.42) | 1.02 (0.87 - 1.40) | 0.631 |
|  | *Ruminococcus* | 1.54 ± 0.46 | 1.72 ± 0.21 | 0.402 |

Note: Data that do not conform to a normal distribution were represented by the median and quartiles. The sample size of each group is 6 (n = 6).

**Table S3 The α-diversity of gut microbiota in rats**

| Index | WH | WL | *F* | *P* |
| --- | --- | --- | --- | --- |
| Obs | 7863.500 ± 246.072 | 7630.667 ± 125.296 | 2.138 | 0.066 |
| Shannon | 4.212 ± 0.111 | 4.201 ± 0.069 | 1.513 | 0.834 |
| Simpson | 0.938 ± 0.012 | 0.940 ± 0.010 | 0.424 | 0.769 |
| Pielou | 0.470 ± 0.118 | 0.470 ± 0.007 | 1.723 | 0.954 |
| Chao1 | 8414.658 ± 289.146 | 8217.063 ± 134.716 | 3.351 | 0.160 |
| ACE | 8308.046 ± 287.112 | 8113.742 ± 135.362 | 2.876 | 0.165 |

Note: The sample size of each group is 6 (n = 6).

**Table S4 The LEfSe and ANCOM-BC results of gut microbiota in rats**

| Species | LEfSe | | | | ANCOM-BC | | | | |
| --- | --- | --- | --- | --- | --- | --- | --- | --- | --- |
|  | Comparison | Group | LDA | p-value | log_2_ FC | q-value | SE | Difference | Significance |
| *s_Lawsonibacter_sp.* | WH - WL | WH | 3.417 | 0.006 | 0.235 | 0.161 | 0.305 | FALSE | Not Significant |
| *s_Helicobacter_typhlonius* | WH - WL | WH | 3.266 | 0.006 | 8.975 | < 0.001 | < 0.001 | TRUE | Enriched in WH |
| *s_Phocaeicola_vulgatus* | WH - WL | WH | 3.164 | 0.006 | 0.58 | 0.012 | 0.044 | TRUE | Not Significant |
| *s_uncultured_Bacteroides_sp.* | WH - WL | WH | 3.106 | 0.016 | 0.365 | 0.082 | 0.187 | FALSE | Not Significant |
| *s_Paramuribaculum_sp.* | WH - WL | WH | 3.086 | 0.004 | 0.236 | 0.073 | 0.171 | FALSE | Not Significant |
| *s_Helicobacter_sp.* | WH - WL | WH | 3.073 | 0.037 | 3.515 | < 0.001 | < 0.001 | TRUE | Enriched in WH |
| *s_Helicobacter_sp._MIT_03-1616* | WH - WL | WH | 2.968 | 0.028 | 8.354 | < 0.001 | < 0.001 | TRUE | Enriched in WH |
| *s_Bacteroides_intestinalis* | WH - WL | WH | 2.953 | 0.004 | 0.808 | 0.002 | 0.012 | TRUE | Not Significant |
| *s_Muribaculum_sp.* | WH - WL | WH | 2.948 | 0.037 | 0.158 | 0.215 | 0.374 | FALSE | Not Significant |
| *s_Akkermansia_muciniphila* | WH - WL | WH | 2.928 | 0.004 | 3.687 | < 0.001 | < 0.001 | TRUE | Enriched in WH |
| *s_Candidatus_Amulumruptor_caecigallinarius* | WH - WL | WH | 2.9 | 0.004 | 1.666 | < 0.001 | < 0.001 | TRUE | Enriched in WH |
| *s_Alphaproteobacteria_bacterium* | WH - WL | WH | 2.821 | 0.025 | 0.282 | 0.19 | 0.343 | FALSE | Not Significant |
| *s_Flintibacter_muris* | WH - WL | WH | 2.702 | 0.01 | 0.072 | 0.624 | 0.763 | FALSE | Not Significant |
| *s_uncultured_Helicobacter_sp.* | WH - WL | WH | 2.696 | 0.004 | 4.039 | < 0.001 | < 0.001 | TRUE | Enriched in WH |
| *s_Acholeplasmatales_bacterium* | WH - WL | WH | 2.647 | 0.01 | 0.646 | 0.019 | 0.063 | FALSE | Not Significant |
| *s_Acutalibacter_sp.* | WH - WL | WH | 2.576 | 0.006 | 0.316 | 0.108 | 0.228 | FALSE | Not Significant |
| *s_Desulfovibrionaceae_bacterium_LT0009* | WH - WL | WH | 2.448 | 0.004 | 4.253 | < 0.001 | < 0.001 | TRUE | Enriched in WH |
| *s_Colidextribacter_sp._OB.20* | WH - WL | WH | 2.423 | 0.037 | 0.026 | 0.878 | 0.932 | FALSE | Not Significant |
| *s_Anaerotignum_sp.* | WH - WL | WH | 2.418 | 0.004 | 1.033 | < 0.001 | < 0.001 | TRUE | Enriched in WH |
| *s_Prevotella_hominis* | WH - WL | WL | -2.69 | 0.025 | -2.227 | < 0.001 | 0.001 | TRUE | Enriched in WL |
| *s_Limosilactobacillus_reuteri* | WH - WL | WL | -2.759 | 0.01 | -1.813 | < 0.001 | < 0.001 | TRUE | Enriched in WL |
| *s_Bacterium_D16-59* | WH - WL | WL | -2.804 | 0.016 | -1.087 | < 0.001 | < 0.001 | TRUE | Enriched in WL |
| *s_Clostridium_sp.* | WH - WL | WL | -2.894 | 0.037 | -0.654 | < 0.001 | < 0.001 | TRUE | Not Significant |
| *s_Eubacterium_plexicaudatum* | WH - WL | WL | -2.901 | 0.004 | -1.469 | < 0.001 | < 0.001 | TRUE | Enriched in WL |
| *s_Ruminiclostridium_sp.* | WH - WL | WL | -2.966 | 0.01 | -1.180 | < 0.001 | < 0.001 | TRUE | Enriched in WL |
| *s_Lachnospira_sp.* | WH - WL | WL | -3.002 | 0.004 | -1.608 | < 0.001 | < 0.001 | TRUE | Enriched in WL |
| *s_Eubacterium_siraeum* | WH - WL | WL | -3.332 | 0.006 | -1.631 | 0.008 | 0.033 | TRUE | Enriched in WL |
| *s_Alistipes_sp.* | WH - WL | WL | -3.375 | 0.01 | -1.991 | < 0.001 | < 0.001 | TRUE | Enriched in WL |
| *s_Eubacterium_sp.* | WH - WL | WL | -3.472 | 0.01 | -0.845 | < 0.001 | < 0.001 | TRUE | Not Significant |
| *s_Bacterium_D16-36* | WH - WL | WL | -3.566 | 0.037 | -1.920 | 0.002 | 0.012 | TRUE | Enriched in WL |

Note: The sample size of each group is 6 (n = 6).
